# Influenza Sentinel Surveillance among Patients with Influenza-Like-Illness and Severe Acute Respiratory Illness within the Framework of the National Reference Laboratory, Niger, 2009-2013

**DOI:** 10.1371/journal.pone.0133178

**Published:** 2015-07-31

**Authors:** Halima Boubacar Maïnassara, Adamou Lagare, Stefano Tempia, Ali Sidiki, Bassira Issaka, Bibata Abdou Sidikou, Odile Ouwe Missi Oukem-Boyer

**Affiliations:** 1 Centre de Recherche Médicale et Sanitaire (CERMES), Niamey, Niger; 2 Influenza Division, Centers for Disease Control and Prevention, Atlanta, Georgia, United States of America; 3 Influenza Division, Centers for Disease Control and Prevention, Pretoria, South Africa; University of Calgary & ProvLab Alberta, CANADA

## Abstract

**Background:**

Little is known about the epidemiology of influenza in Africa, including Niger. We documented the epidemiology of seasonal and pandemic influenza among outpatients with influenza-like-illness (ILI) and inpatients with severe acute respiratory illness (SARI) presenting at selected sentinel sites in Niger from April 2009 through April 2013.

**Methods:**

Patients meeting the ILI or the SARI case definitions and presenting at the outpatient or inpatient departments of selected sentinel sites were enrolled. Epidemiological data and nasopharyngeal swabs were collected. The respiratory samples were tested by real-time reverse transcription polymerase chain reaction.

**Results:**

From April 2009 to April 2013, laboratory results were obtained from 1176 ILI and 952 SARI cases, of which 146 (12%) and 54 (6%) tested positive for influenza virus, respectively. The influenza positivity rate was highest in the 5-14 year age-group (32/130; 24% among ILI patients and 6/61; 10% among SARI patients) followed by the 1-4 year age-group (69/438; 16% among ILI patients and 32/333; 9% among SARI patients). Of the 200 influenza positive cases 104 (52%) were A(H1N1)pdm09, 62 (31%) were A(H3N2) and 34 (17%) were B. Influenza viruses were detected predominantly from November to April with peak viral activity observed in February.

**Conclusions:**

The Niger sentinel surveillance system allowed to monitor the circulation of seasonal influenza as well as the introduction and spread of influenza A(H1N1)pdm09 in the country. Continuous influenza surveillance is needed to better understand the epidemiology of seasonal influenza and monitor the emergence of influenza strains with pandemic potential.

## Introduction

Globally, pneumonia is among the leading causes of mortality [1] with the highest burden experienced in sub-Saharan Africa and Asia [2]. Influenza virus infection is a common cause of pneumonia and is responsible for an estimated 3–5 million cases of severe illness and 250,000–500,000 deaths worldwide every year [3]. Individuals <5 and ≥65 years of age experienced the highest burden [4,5,6].

The epidemiology of influenza has been well characterized in temperate countries of the northern and southern hemisphere, while, until recently, the burden of influenza virus infection in Africa and particularly in sub-Saharan Africa was poorly understood. A review of surveillance data in 15 African countries from 2006 to 2010 reported that approximately 10% of severe acute respiratory illness (SARI) hospitalizations and 25% of influenza-like-illness (ILI) outpatient visits were associated with influenza virus infection [7]. Nonetheless, data on the disease burden associated with influenza virus infection remain scarce in several African countries, including Niger.

In 2009, the Government of Niger established a national influenza surveillance system aiming at monitoring the circulation of seasonal influenza as well as detecting the emergence and spread of novel influenza strains with pandemic potential. In its capacity of National Reference Laboratory for Influenza (NLRI), the Centre de Recherche Médicale et Sanitaire (CERMES) is mandated to provide laboratory diagnosis for influenza virus infection, participate to the training of laboratory technicians, supervise other laboratories, inform the Ministry of Public Health about any unusual situation related to influenza virus activity and provide regular reports. In this study we report the epidemiology of seasonal and pandemic influenza (A(H1N1)pdm09) among outpatients with influenza-like-illness (ILI) and inpatients with severe acute respiratory illness (SARI) presenting at selected sentinel sites in Niger from April 2009 to April 2013.

## Materials and Methods

### Ethics and enrollment procedures

In 2009, the National Ethics Committee of Niger (CCNE) authorized the Centre de Recherche Médicale et Sanitaire (CERMES) to start human influenza surveillance (reference number 06/2009/CCNE), and in 2011, CERMES became the National Influenza Reference laboratory (reference number 249 MSP/DGSP/DPHL/MT). In 2012, the CCNE provided an extended approval for human influenza surveillance (reference number 020/2012/CCNE). All cases that met ILI or SARI diseases definitions were eligible for enrollment. Verbal informed consent was obtained from all the patients (cases who were 18 years of age and older) because the majority of them were not educated to read themselves the consent. So physicians explained them the study and asked them verbally if they accepted to be enrolled. We did not document the consent. Those who did not accept were not included. Proxy informed and verbal consent was obtained from parents or legal guardians of minors after explanation of the study by the physicians. Consent was verbal because majority of parents were not educated enough to read and signed the consent paper themselves. Patients who did not meet the case definition or did not provide verbal consent were not included. These consent procedures were approved by the ethics committee. A standardized questionnaire was administrated by clinical personal, to record patients’ demographic characteristics and medical history. The questions included information on date of enrolment and symptom onset, gender, age and clinical symptoms. In very young children (< 3 years of age), specific symptoms (e.g. sore throat, headache and myalgia) could not be properly ascertained. In 2012, the CCNE which is Niger institutional ethics committee provided an extended approval for human influenza surveillance and this study (reference number 020/2012/CCNE).

### Study design and setting

Niger is a large land-locked country situated in West Africa, characterized by an arid climate. Four seasons can be distinguished in Niger: the cold season (from mid-December to mid-February) with temperature ranging between 19 and 27°C; the dry and hot season (from mid-February to May) with temperature ranging between 28 and 33°C; the rainy season (from June to September) with temperature ranging between 28 and 32°C; and the hot season (from October to mid-December) with mean temperature of 35°C (Directorate of National Meteorology, personal communication).

**Fig 1:**
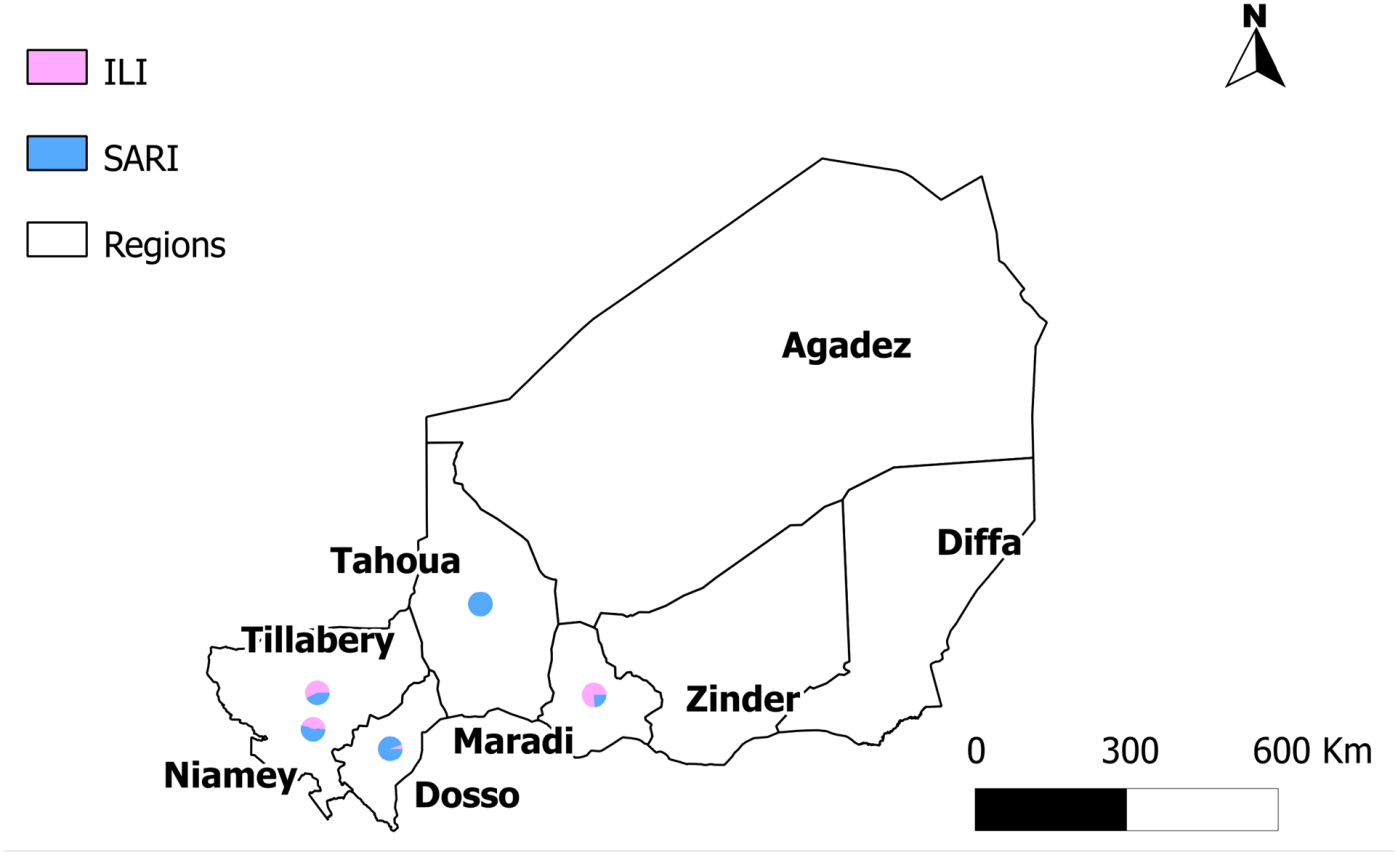
Location of sentinel surveillance sites and proportion of enrolled severe acute respiratory illness (SARI) and influenza-like illness (ILI) cases, Niger, 2009–2013.

We conducted prospective surveillance from April 2009 to April 2013 in 8 sentinel sites located in 5 of the 8 regions of the country (Fig 1). In 2012 the population residing in the 5 regions where influenza sentinel surveillance was implemented represented 75.9% (13,010,000 people) of the country population (17,129,076 people) [8]. The sentinel sites comprised 7 public hospitals (where surveillance was implemented in the outpatients and inpatients departments) and 1 outpatient clinic where only outpatients were enrolled. Three sentinel sites located in Niamey started surveillance in April 2009 (Hôpital National, Centre Hospitalier Universitaire Lamordé and Clinique Pasteur). The sentinel surveillance network was subsequently expanded with the addition of 4 sentinel sites located in 4 Regions as follows: Maradi Region (Centre Hospitalier Régional de Maradi) in September 2009, Dosso Region (Hôpital de District de Gaya), Tillabery Region (Hôpital de District de Tillabéry) in November 2009 and Tahoua Region (Centre Hospitalier Régional de Tahoua) in February 2013. The Centre Hospitalier Régional Poudrière of Niamey started surveillance in March 2010.

An ILI case was defined as an outpatient with sudden onset of fever (>38°C) and cough or sore throat, with the onset of symptoms within 7 days prior to the presentation at the health facility [9,10]. A SARI case in children <5 years of age was defined as an inpatient with cough or difficulty breathing within 7 days prior to hospital admission, and at least one danger signs (unable to drink or breastfeed, lethargic, vomits everything, convulsions, nasal flaring, chest indrawing, stridor in a calm child or tachypnea). For patient ≥5 years of age, a SARI case was defined as an inpatient with fever (≥38°C), cough and shortness of breath or difficulty breathing within the 7 days prior to hospital admission [9, 10].

A standardized questionnaire was administrated by clinical personnel to record patients’ demographic characteristics and medical history. The questions included information on date of enrolment and symptom onset, gender, age and clinical symptoms. In very young children (< 3 years of age), specific symptoms such as sore throat, headache and myalgia could not be properly ascertained.

### Sample collection and laboratory procedures

Nasopharyngeal swabs were collected from all enrolled cases and placed in cryovials containing virus transport medium (Copan kit). The specimens were kept refrigerated at 4°C at the sentinel site and then transported twice per week to the NRLI for testing. Samples were first screened for influenza A and B viruses by real-time reverse transcription polymerase chain reaction (rRT-PCR). Specimens found to be positive for influenza A virus were subtyped using a RT-PCR assay with subtype-specific primers [11].

### Data management and analysis

Questionnaire information and laboratory results were recorded in a central database kept at the CERMES Epidemiology Unit. We analyzed the demographic characteristics of the study subjects and the positive cases, as well as the seasonal patterns of influenza virus circulation using Stata version 12 (StataCorp, Texas, USA). We used the *X* ^*2*^ test or fisher exact test to assess differences in proportion. A two-sided p-value of <0.05 was considered significant.

## Results

### Characteristic of enrolled patients

Over the study period we enrolled 2128 patients, of which 1176 (55%) were ILI and 952 were SARI cases (Table 1). There were 1147 (54%) males and 1383 (65%) patients were enrolled from the sentinel sites situated in Niamey. There was a statistically significant difference in the proportion of males enrolled among SARI and ILI cases (59% vs 50% respectively, p <0.001). Among cases with known age (n = 2110; 99%) the patient’s age ranged between 2 days and 90 years (median age 1.5 years) and children aged <5 years accounted for 70% (817/1164) and 83% (788/946) of the total number of ILI and SARI cases, respectively. Individuals ≥65 years of age accounted for 0.8% (10/1164) of the ILI and 2.4% (23/946) of the SARI cases enrolled. The number of enrolled ILI and SARI cases gradually increased during the study period with the higher number of cases enrolled in 2011 and 2012. There was a statistically significant variation of the annual number of enrolled SARI and ILI (p < 0.001).

**Table 1:** Characteristics of influenza-like illness (ILI) and severe acute respiratory illness (SARI) cases, Niger, 2009–2013.

Characteristics	ILI	SARI	P—value
	N = 1176	N = 952	
	n (%)	n (%)	
**Age (years)**			p < 0.001
< 1	379 (32)	455 (48)	
1–4	438 (37)	333 (35)	
5–14	130 (11)	61 (6)	
15–29	85 (7)	20 (2)	
≥30	132 (11)	77 (8)	
Missing	12 (1)	6 (< 1)	
**Sex**			p < 0.001
Male	588 (50)	559 (59)	
Female	588 (50)	393 (41)	
**Year**			p < 0.001
2009	128 (11)	1 (< 1)	
2010	298 (25)	88 (9)	
2011	429 (36)	429 (45)	
2012	253 (22)	373 (39)	
2013	67 (6)	61 (6)	
Missing	1 (< 1)	0 (0)	
**Seasons**			p = 0.007
Hot (October to mid-December)	189 (16)	208 (22)	
Cold (Mid-December to mid-February)	180 (15)	132 (14)	
Rain (June to September)	280 (23)	227 (23)	
Dry (Mid-February to May)	526 (45)	385 (40)	
Missing	1 (< 1)	0 (0)	

The p—value is for the comparison of the characteristics of patients with ILI and patients with SARI

### Detection of influenza virus

Laboratory results were obtained from all 1176 ILI and 952 SARI cases, of which 146 (12%) and 54 (6%) tested positive for influenza virus, respectively (p<0.001). The proportion of samples testing positive for influenza viruses was highest in the 5–14 year age-group (32/130; 25% among ILI patients and 6/61; 10% among SARI patients) followed by the 1–4 year age-group (69/438; 16% among ILI patients and 32/333; 9% among SARI patients) (Table 2). Conversely, the proportion of samples testing positive for influenza viruses was lowest in the <1 year age-group (20/379; 5% among ILI cases and 11/455; 2% among SARI cases). Among patients with available duration of symptoms (2095/2128; 98.5%), there was no statistically significant difference in the influenza detection rate among specimens collected with 3 days of symptoms onset and those collected after 3 days (132/1452: 9.1% vs. 64/643: 9.9%; p = 0.983).

**Table 2:** Number (and percent) of samples tested positive for influenza virus among influenza-like illness (ILI) and severe acute respiratory illness (SARI) cases, Niger, 2009–2013.

	ILI			SARI			P—value
Characteristics	Influenzae A	Influenzae B	Total	Influenzae A	Influenzae B	Total	
	n/N (%)	n/N (%)	n/N (%)	n/N (%)	n/N (%)	n/N (%)	
**Age (years)**							p = 0.182
< 1	20/379 (5)	0/379 (0)	20/379 (5)	7/455 (1)	4/455 (1)	11/455 (2)	
1–4	55/438 (13)	14/438 (3)	69/438 (16)	28/333 (1)	4/333 (1)	32/333 (9)	
5–14	25/130 (19)	7/130 (6)	32/130 (25)	5/61 (8)	1/61 (2)	6/61 (10)	
15–29	9/85 (11)	2/85 (13)	11/85 (13)	1/20 (5)	0/20 (0)	1/20 (5)	
≥30	11/132 (8)	1/132 (1)	12/132 (9)	3/77 (4)	0/77 (0)	3/77 (4)	
**Sex**							p = 0.732
Male	57/588 (10)	13/588 (2)	70/588 (12)	27/559 (5)	5/559 (1)	32/559 (6)	
Female	64/588 (11)	12/588 (2)	76/588 (13)	18/393 (5)	4/393 (6)	22/393 (6)	
**Seasons**							p = 0.039
Hot	5/189 (3)	2/189 (1)	7/189 (4)	7/208 (3)	0/208 (0)	7/208 (3)	
Cold	45/180 (25)	4/180 (2)	49/180 (27)	13/132 (10)	5/132 (4)	18/132 (14)	
Rain	21/280 (7)	1/280 (1)	22/280 (8)	2/227 (1)	0/227 (0)	2/227 (1)	
Dry	50/526 (10)	18/526 (13)	68/526 (13)	23/385 (1)	4/385 (1)	27/385 (7)	

**٭** Comparison of the influenza detection rate among patients with ILI and patients with SARI

The p—value is for the comparison of the influenza detection rate among patients with ILI and patients with SARI

Of the 200 influenza positive samples 104 (52%) were influenza A(H1N1)pdm09, 62 (31%) were influenza A(H3N2) and 34 (17%) were influenza B. No influenza A and B virus co-infection was detected.

### Seasonality and circulation of influenza types and subtypes

**Fig 2:**
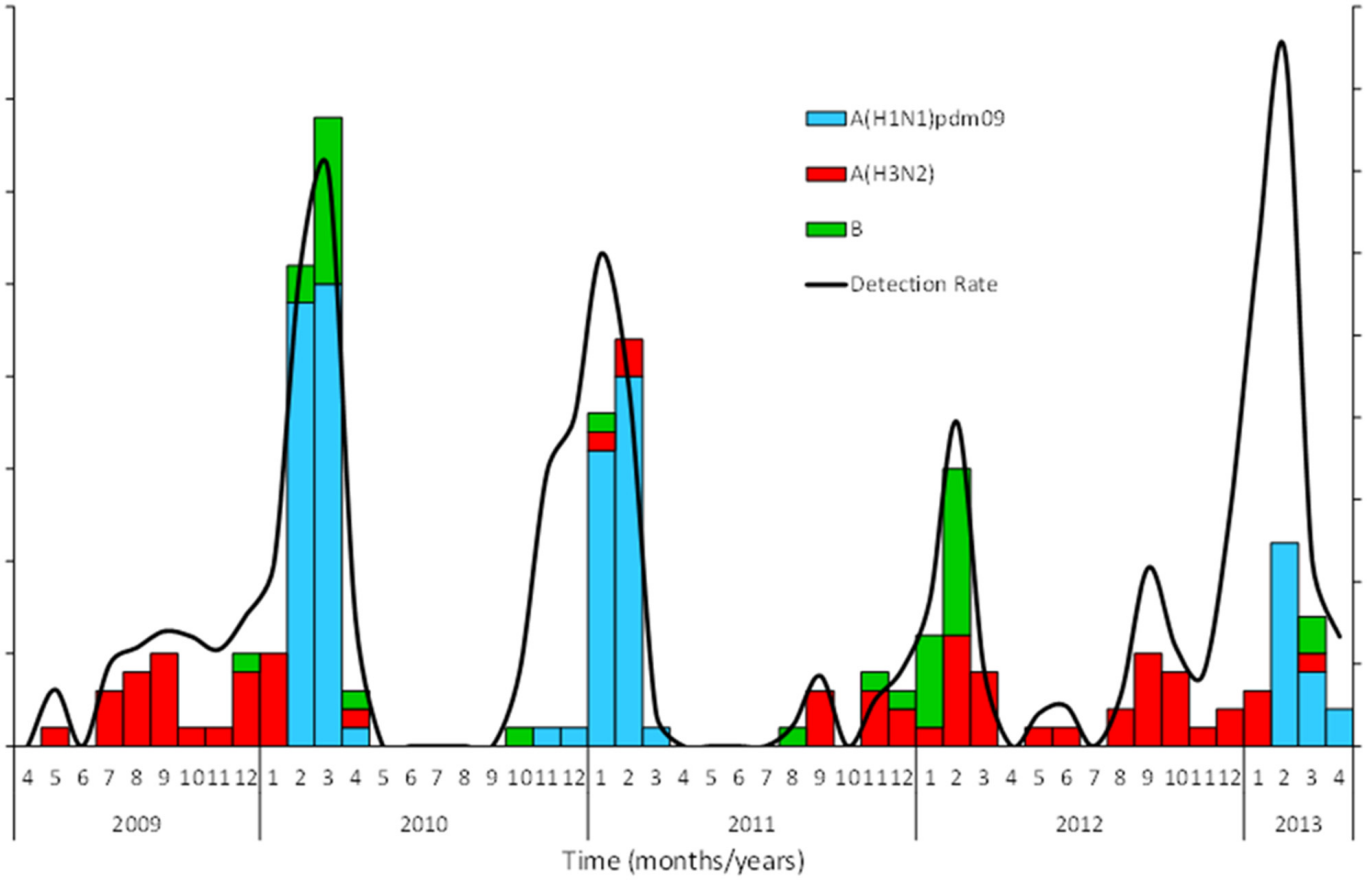
Number and percent of samples tested positive for influenza viruses by month, Niger, 2009–2013.

Influenza virus was detected predominantly from November to April (169/200; 84.5%), which corresponds to the cold and dry seasons, with peak viral activity observed in February (Fig 2). Influenza A(H3N2) was the dominant circulating subtype from November to December 2009. Influenza A(H1N1)pdm09 was first detected in Niger in January 2010 and became the dominant subtype during the 2009–2010 and 2010–2011 influenza seasons. Conversely, influenza A(H3N2) and B co-circulated during the 2011–2012 and 2012–2013 influenza seasons followed by A(H1N1)pdm09 from February 2013.

## Discussion

Influenza viruses were detected in both ILI and SARI cases and the burden was highest among children (1–14 years).

The overall influenza detection in our setting among ILI and SARI cases was comparable with those reported in other African countries [7]. Surveillance data from 15 African countries from 2006–2010 also reported higher detections of influenza virus in children <15 years of age for both SARI and ILI compared to older age groups [7].

In our settings, influenza virus circulation was mostly during the cold and dry seasons from November to April. The influenza seasonality observed in Niger differs from those observed in other countries in West Africa, like Senegal, Ivory Coast, Ghana and Nigeria, where influenza virus circulation was detected with increased activity during the rainy season [7, 12, 13, 14, 15]. The influenza seasonal patterns in Niger mimic more closely those observed in Morocco [16] and the temperate regions of the northern hemisphere.

In Niger, elevated numbers of influenza A(H1N1)pdm09 virus were observed only from February 2010, when the pandemic virus replaced the circulating influenza A(H3N2) subtype. This is in contrast to the majority of European, Eastern and Southern Africa countries [17], where significant increases in numbers of newly reported cases were observed beginning in July 2009. Nonetheless, delayed community transmission of influenza A(H1N1)pdm09 (toward the end of 2009 or the beginning of 2010) was observed in other countries of West, Central and Northern Africa, including Cameroon, Cape Verde, Ghana, Guinea, Ivory Coast, Mali, Mauritania, Morocco, Nigeria, and Senegal [18,19].

The majority of the enrolled SARI and ILI cases were children <5 years of age reflecting the high burden of respiratory illness in this age group. Nonetheless, the high proportion of children enrolled in our study can also be attributed to a differential healthcare seeking behavior between age groups in Niger as well as difficulties experienced in the enrollment of older persons.

The influenza detection rate was not statistically different among patients with symptoms onset ≤3 and ˃3 days indicating that influenza virus can still be detected at higher rates even in patients with prolonged duration of symptoms.

Our study has limitations that warrant discussion. First, we established sentinel surveillance only in 5/8 Regions of the country and 65% of patients were enrolled in sentinel sites situated in Niamey. This could potentially affect the generalizability of our results to the entire population. Nonetheless, the population of the Regions where sentinel surveillance was established represented approximately 76% of the population of Niger in 2012 [8]. Second, while the timing of the influenza season in Niger would allow the use of the Northern Hemisphere influenza vaccine no characterization of the influenza virus strains was available for the study period hindering the ability to assess the matching of influenza virus strains circulating in Niger with the composition of the annual influenza vaccine for the Northern Hemisphere. No influenza vaccination is currently implemented in Niger. Third, we did not keep formal records of the proportion of patients consenting to participate in the study. However, a review of the performance of the surveillance system implemented through hospital record review at sentinel sites revealed that only few patients that met the study case definition were missed by the surveillance program. In conclusion, influenza virus infection was associated with both mild and severe disease in Niger. In our settings however, influenza prevention and control interventions compete with those of other prevalent diseases such as malaria, diarrhea and malnutrition. The current surveillance system should be reinforced, extended and strengthened to continuously monitor seasonal influenza virus activity in the country as well as to early detect the emergence or introduction of novel influenza strains with pandemic potential. In addition, further studies aiming at assessing the influenza-associated burden as well as high-risk groups for influenza-associated severe illness are needed in our settings to advocate for targeted interventions.
